# Invasive *Phragmites australis* management outcomes and native plant recovery are context dependent

**DOI:** 10.1002/ece3.5820

**Published:** 2019-12-05

**Authors:** Christine B. Rohal, Chad Cranney, Eric L. G. Hazelton, Karin M. Kettenring

**Affiliations:** ^1^ Department of Watershed Sciences and the Ecology Center Utah State University Logan UT USA; ^2^ Department of Environmental Horticulture University of Florida Gainesville FL USA; ^3^ Utah Division of Wildlife Resources Salt Lake City UT USA

**Keywords:** contingency, herbicide, invasive plant management, *Phragmites australis*, Utah

## Abstract

The outcomes of invasive plant removal efforts are influenced by management decisions, but are also contingent on the uncontrolled spatial and temporal context of management areas. *Phragmites australis* is an aggressive invader that is intensively managed in wetlands across North America. Treatment options have been understudied, and the ecological contingencies of management outcomes are poorly understood. We implemented a 5‐year, multi‐site experiment to evaluate six *Phragmites* management treatments that varied timing (summer or fall) and types of herbicide (glyphosate or imazapyr) along with mowing, plus a nonherbicide solarization treatment. We evaluated treatments for their influence on *Phragmites* and native plant cover and *Phragmites* inflorescence production. We assessed plant community trajectories and outcomes in the context of environmental factors. The summer mow, fall glyphosate spray treatment resulted in low *Phragmites* cover, high inflorescence reduction, and provided the best conditions for native plant recruitment. However, returning plant communities did not resemble reference sites, which were dominated by ecologically important perennial graminoids. Native plant recovery following initial *Phragmites* treatments was likely limited by the dense litter that resulted from mowing. After 5 years, *Phragmites* mortality and native plant recovery were highly variable across sites as driven by hydrology. Plots with higher soil moisture had greater reduction in *Phragmites* cover and more robust recruitment of natives compared with low moisture plots. This moisture effect may limit management options in semiarid regions vulnerable to water scarcity. We demonstrate the importance of replicating invasive species management experiments across sites so the contingencies of successes and failures can be better understood.

## Graphical abstract

Managers of North American wetlands seek practical and effective methods for removing the invasive plant, *Phragmites australis*, and restoring invaded wetlands to native plant communities. At six environmentally variable sites within Great Salt Lake, Utah, we evaluated six *Phragmites* management treatments that primarily varied timing and types of herbicide along with mowing. Summer mowing combined with fall herbicide application resulted in the best combination of low *Phragmites* cover, reduction in *Phragmites* inflorescences, and an increase in native plant recruitment; however, all herbicide treatments had highly variable plant community outcomes across sites, highlighting the context‐dependency of vegetation recovery success following *Phragmites* management.

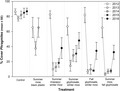

## 1 INTRODUCTION

The outcomes of ecosystem restoration following invasive species management are highly influenced by spatial and temporal contingencies (Stewart et al., 2008; Suding, 2011). Therefore, making usable management prescriptions is often impossible without detailed analysis of these contingencies. The broad range of many plant invasions, a result of the generalist nature of many invasive species, can lead to divergent outcomes of even the same treatment across ecological situations, particularly in native plant recovery following management. The factors attributed to variable results in removal experiments are diverse from broad‐scale climatic differences (Le Duc, Pakeman, Putwain, & Marrs, 2000) to small‐scale patterns in soil conditions (Eviner & Hawkes, 2008). But restoration failures are often attributed to problematic sites, instead of being critically assessed for why management responses are variable across sites, and incorporating lessons learned from this variability into management prescriptions (Eviner & Hawkes, 2008).

Multi‐site experiments can help researchers understand the context dependencies in the success of invasive plant removal efforts (Cox, Marrs, Pakeman, & Duc, 2008; Stewart et al., 2008), yet many previous invasive plant management studies have taken place in mesocosms or a single experimental field, limiting our understanding of the environmental influences on treatment outcomes (Flory, 2010; Kettenring & Adams, 2011). By expanding the geographic range of sites that receive the same management regime, researchers can link constraints, such as abiotic conditions, land‐use history, and landscape setting, with trajectories following invasive plant management (Suding, 2011). These multi‐site experiments across many possible restoration contexts can help managers prioritize sites that are more likely to reach restoration goals following the removal of the invader. Multi‐site experiments can also help identify possible environmental thresholds that may prevent successful invader control or native plant recovery. Managers can limit cost‐intensive management activities at sites that reach these identified thresholds, or plan for more intensive intervention at locations that have more constraints (Suding, 2011).

One invasive plant that is of great concern to land managers across North America is *Phragmites australis* (Cav.) Trin ex Steud (common reed, hereafter called *Phragmites*), a widespread wetland grass with a global distribution (Eller et al., 2017; Kettenring, de Blois, & Hauber, 2012). While there is a native *Phragmites* lineage in North America that is not invasive, it is the introduction of an invasive lineage from Eurasia that has led to the rapid expansion of *Phragmites* into coastal and inland wetlands (Saltonstall, 2002). A primary concern is invasive *Phragmites*' ability to outcompete native vegetation (Meyerson, Saltonstall, Windham, Kiviat, & Findlay, 2000) and displace habitat leading to declines in floral and faunal biodiversity (Dibble, Pooler, & Meyerson, 2013). *Phragmites* has been the focus of large‐scale management efforts and some management research, particularly in the northeast United States (reviewed in Hazelton, Mozdzer, Burdick, Kettenring, & Whigham, 2014). But despite large financial investments in its management (Martin & Blossey, 2013), quantitative evidence for the effectiveness of *Phragmites* management efforts, particularly in their capacity to meet the goal of restoration to native plant communities, is lacking (Hazelton et al., 2014; Zimmerman, Shirir, & Corbin, 2018). *Phragmites* thrives across wide environmental gradients within wetlands, particularly under high nitrogen conditions where its growth is more robust and it produces more inflorescences (Kettenring, McCormick, Baron, & Whigham, 2011; Meyerson, Cronin, & Pyšek, 2016; Mozdzer & Zieman, 2010), but most management studies have been conducted across few replicate sites (Hazelton et al., 2014), which limits our understanding of the context dependencies of treatment responses. Past studies were also short in duration (commonly 3 years or less) and done at spatial scales too small to be highly relevant to managers who work across environmentally variable landscapes (Hazelton et al., 2014).

Herbicide is the most widespread tool used for *Phragmites* management, with glyphosate the most common, and imazapyr a more recent, but more expensive option (Martin & Blossey, 2013). Others have sought nonchemical *Phragmites* management options to minimize environmental impacts, like solarization (heating soil to temperatures lethal to *Phragmites* rhizomes), but these strategies have not been rigorously evaluated. Managers have expressed uncertainties about the most effective type of herbicide, as well as the best timing of herbicide application for both *Phragmites* removal and native plant recovery (Rohal, Kettenring, Sims, Hazelton, & Ma, 2018). Imazapyr was more effective than glyphosate at *Phragmites* removal in some studies (Derr, 2008; Mozdzer, Hutto, Clarke, & Field, 2008), but there are questions about its long‐term impact on native plant recovery due to its persistence (up to 4 years) in the soil, and ability to be adsorbed by plant roots (Hazelton et al., 2014; Tu, Hurd, & Randall, 2001). Summer applications of herbicide were equally, if not more, effective than fall applications at *Phragmites* removal in short‐term management studies with limited monitoring (e.g., 1–2 growing seasons of treatment with post‐treatment monitoring in year of or just 1 year post‐application; Derr, 2008; Mozdzer et al., 2008). In addition, questions remain about the long‐term influence of herbicide application timing on *Phragmites* and native plants. Herbicide application in the fall when native plants senesce may have fewer nontarget plant impacts (Hazelton et al., 2014). However, summer instead of fall applications may be more effective in locations that frequently become drought stressed in late summer and fall (Meyerson, Lambert, & Saltonstall, 2010). Counterintuitively, herbicide is less effective when sprayed on plants that are stressed, because herbicides work by attacking metabolic processes, which are often shut down when plants are stressed (Tu et al., 2001). In semiarid regions of North America where *Phragmites* is rapidly expanding its range (Kettenring et al., 2012; Long, Kettenring, Hawkins, & Neale, 2017), low water availability later in the growing season may limit herbicide effectiveness. Managers often use mowing to reduce the dead *Phragmites* biomass that impedes native plant recruitment, particularly where burning for biomass management is infeasible due to air quality and safety concerns, such as areas near major population centers like Salt Lake City. But the timing of mowing (before or after herbicide application) may have implications for herbicide effectiveness and litter degradation speed. The timing of herbicide application and mowing may also influence *Phragmites* inflorescence production, a critical management concern because invasive *Phragmites* reproduces prolifically by seed (Kettenring & Mock, 2012).

Given these uncertainties in *Phragmites* management, we developed a multi‐site, large‐plot, and multi‐year experiment. We addressed these questions: First, what are effective treatments for reducing *Phragmites* cover? We expected that our results would mirror other studies—imazapyr applications would be superior to glyphosate, and summer applications would be superior to fall applications (Derr, 2008; Mozdzer et al., 2008). Second, which treatments limit *Phragmites* seed production? We hypothesized that the summer treatments might prevent seed production relative to the fall due to the earlier herbicide application limiting seed production. Third, what are treatment impacts on native plants? We expected imazapyr treatments to negatively influence native plants, due to this herbicide's purported ability to persist in the soil (Hazelton et al., 2014). We also expected summer herbicide applications to negatively influence native plants compared with fall applications, due to a greater likelihood for nontarget plant mortality. Finally, how do environmental factors influence treatment effectiveness? We hypothesized that (a) *Phragmites* cover following treatments will be higher in nutrient enriched areas, while native species recovery would be less robust, due to greater growth of and competition with *Phragmites*, (b) areas that experience drought will have less effective *Phragmites* removal with herbicide.

## 2 METHODS

### 2.1 Study sites

**Figure 1:**
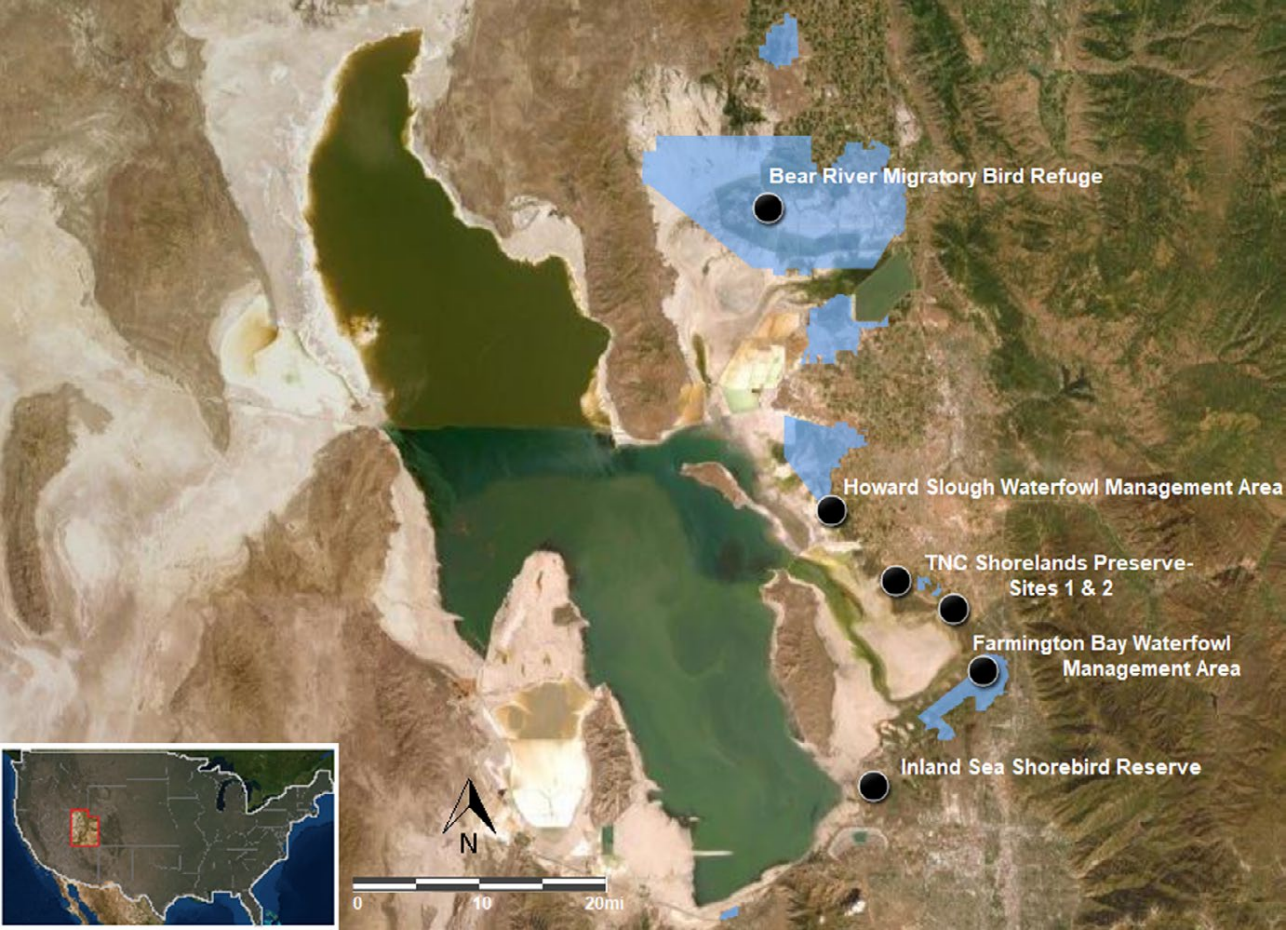
Map of six experimental *Phragmites* management sites in Great Salt Lake, Utah. Sites include U.S. Fish & Wildlife Services: Bear River Migratory Bird Refuge (BR); Utah Division of Wildlife Resources: Howard Slough Waterfowl Management Area (HS) and Farmington Bay Waterfowl Management Area (FB); The Nature Conservancy: Shorelands Preserve (two separate locations TN and TS); and Kennecott Utah Copper: Inland Sea Shorebird Reserve (IS). Figure courtesy of Emily Leonard

This study was conducted in six wetland sites on the eastern shore of the Great Salt Lake, Utah, and included broad representation of Great Salt Lake land owners (Figure 1) to ensure research relevance to management. Our aim was to select sites that represented a gradient in environmental conditions in order to assess the influence of a wide range of environmental factors that are common in inland wetlands, particularly variability in hydrology, salinity, and nutrient enrichment. Hydrologic conditions ranged from drought prone, to hydrologically managed wetlands with consistent flooding (man‐made impoundments are a very common tool in arid wetlands for mitigating water scarcity), to unaltered hydrology with a typical spring peak but persistent moisture through summer (Downard, Endter‐Wada, & Kettenring, 2014; Long et al., 2017). Nutrient conditions ranged from wetlands with known eutrophic water sources to more isolated wetlands with fewer sources of nutrient enrichment (Table S1). Although Great Salt Lake wetlands are unique with respect to their location in the semiarid Intermountain West of North America, the hydrologic, salinity, and nutrient gradients and native plant communities are representative of wetlands in other regions of *Phragmites*' introduced range (e.g., New England and mid‐Atlantic brackish wetlands with a strong anthropogenic influence).

Dominant native vegetation includes *Typha domingensis*/*T. latifolia* (cattails), *Bolboschoenus maritimus* (alkali bulrush), *Schoenoplectus acutus* (hardstem bulrush), *S. americanus* (common threesquare), and *Distichlis spicata* (saltgrass; Downard, Frank, Perkins, Kettenring, & Larese‐Casanova, 2017). *Phragmites* began to invade Great Salt Lake wetlands after floods in the 1980s (Kettenring et al., 2012; Rohal et al., 2018) and now occupies more than 93 km^2^ in the region (Long et al., 2017).

### 2.2 Experimental design

We established five 20 m × 50 m experimental permanent plots containing dense *Phragmites* at each site, placed at least 20 m apart to avoid herbicide drift between plots and to ensure negligible belowground rhizome connectivity between treatment areas. Plot locations were ≥75% *Phragmites* cover, unmanaged for at least 5 years, and accessible by managers and their equipment. We established one 20 m × 50 m reference (“REF”) plot in native vegetation at each site (*n* = 6) that best represents a target plant community. We selected target plant communities following interviews with property resource managers, who considered their assessment of typical hydrology, nearby vegetation, and previous vegetation in that area (if known).

We evaluated six *Phragmites* management treatments: (a) summer glyphosate spray, followed by a winter mow (“SGWM”); (b) summer imazapyr spray, followed by a winter mow (“SIWM”); (c) fall glyphosate spray, followed by a winter mow (“FGWM”); (d) summer mow, followed by a fall glyphosate spray (“SMFG”); (e) summer mow then cover plots with heavy‐duty black plastic (i.e., a solarization treatment; “SMBP”); and (f) untreated control (“CONT”). These treatments were chosen based on manager interest and feasibility after lengthy interviews and a formal survey (Rohal et al., 2018). We randomly assigned each treatment to a plot (*n* = 30) at five of six sites, such that all six treatments were replicated five times in a randomized, balanced, incomplete block design (Figure S1). This incomplete design was necessary due to imazapyr permitting restrictions at one site and black plastic feasibility restrictions at another.

### 2.3 Treatment application

We applied herbicides initially using equipment that varied with land management partners, but care was taken to ensure equal application rates across sites. In one configuration, a soft‐track wetland tractor (Loglogic) was equipped with a piston‐driven sprayer and a “boomless” nozzle held approximately 3 m above the ground that sprays outward from the back of the vehicle. In another configuration, engine‐powered herbicide hoses were attached to a vehicle (truck, ATV, or Wilco [Wilco Marsh Buggies and Draglines]) from which the plot was hand‐sprayed. Glyphosate (Aquaneat^®^) was applied at a rate of 3 quarts per acre (7 L/ha). Imazapyr (Polaris^®^) was applied at the same rate. Both herbicides were mixed with the nonionic surfactant, LI‐700 at a rate of 1.89 L/378.54 L of mixed solution. We completed follow‐up treatments by spot treating the remaining *Phragmites* shoots using backpack sprayers. We sprayed herbicides on sunny, nonwindy days to minimize herbicide drift, following manufacturers' recommended rates.

We mowed *Phragmites* stems, mulched the biomass (to prevent resprouting from viable nodes in summer and to accelerate decomposition after winter mows), and left the debris on site (since litter removal is rarely feasible for large‐scale management). In low‐water, easy access areas, mowing was conducted using an ASV PT‐80 tracked skid steer (ASV Inc.) with a front‐end hydraulic rotary mower fastened to the front. In deeper water areas, mowing was conducted using a Marsh Master (Coast Machinery LLC) with a hydraulic rotary motor. For the solarization treatment, we placed black plastic (6 mils; 12 m by 30 m rolls) over recently mowed *Phragmites* in July 2012 and secured it until April of the following year (plastic installed for 9 months), when it was permanently removed. We applied all herbicide treatments first in 2012 and conducted follow‐up herbicide treatments in 2013 and 2014. This sequence represents common practice for wetland herbicide application with glyphosate based on manager trial‐and‐error experiences and logistical constraints (C. Cranney, pers. obs.). We conducted mowing treatments in 2012 and 2013. We conducted summer herbicide and mow treatments in early July, fall herbicide treatments in late August, and winter mow treatments in January through March.

### 2.4 Data collection

We monitored vegetation in treatment plots annually starting with pretreatment data in June 2012 and post‐treatment in June 2013–2016. This 5‐year time frame was selected to reflect typical monitoring periods that often accompany funded management sequences and is longer than the 3 years or less of monitoring from most previous *Phragmites* studies (Hazelton et al., 2014). We monitored reference vegetation annually in June 2014–2016. We stopped monitoring the black plastic treatment plots following the 2014 summer due to the rapid return of *Phragmites*, evidence of a failed treatment. Our systematic vegetation sampling design in each plot included four permanent, evenly spaced transects with four, evenly spaced 1 m^2^ quadrats along each transect (Figure S2). We determined percent cover of vegetation by visual estimation in each 1 m^2^ quadrat using modified Daubenmire cover classes (<1%, 1%–5%, >5%–25%, >25%–50%, >50%–75%, >75%–95%, and >95%–100%) using a single observer to ensure consistency. We identified plants to the species level using Flora of Utah (Welsh, Atwood, Higgins, & Goodrich, 1993), and up‐to‐date nomenclature was determined using the USDA PLANTS database (USDA, NRCS, 2019).

For each plot in each sampling period, we determined species richness and adjusted floristic quality assessment index (adjusted FQAI), an evaluation metric that estimates habitat quality. Adjusted FQAI uses a measure of ecological conservatism (mean *C*‐value) and a variant of the FQAI score that considers both the contribution of nonnative species and the intrinsic low species richness of some high‐quality wetlands, like Great Salt Lake (Downard et al., 2017; Miller & Wardrop, 2006). The adjusted FQAI score was calculated as follows:

$$\text{Adjusted}\;\text{FQAI}=\left(\frac{C\sqrt{N}}{10\sqrt{N+A}}\right)\times 100$$

where *C* is the mean *C*‐value, *N* is the number of native species, and *A* is the number of nonnative species per plot. We used *C*‐values developed for other semiarid, Western states and evaluated for applicability in Utah to calculate the mean *C*‐value for each plot in every sampling period (Menuz, Sempler, & Jones, 2016; Table S2).

We collected data on flowering rates (inflorescences per m^2^) by counting all inflorescences in each 1 m^2^ quadrat during the peak of *Phragmites* flowering season each year (except 2015) following all herbicide treatments. In 2014, following the final fall herbicide treatment, we collected and weighed eight inflorescences on each transect at 2 m intervals from each plot to determine seed production and seed viability following the full 3‐year treatment cycle (960 total samples). We then weighed two spikelet subsamples of two representative inflorescences from each plot, from which all florets were counted and averaged by plot. We counted all seeds from each subsample and placed them for 24 hr in a 0.1% tetrazolium solution (Peters, 2000) to determine the number of viable seeds per subsample mass (spikelet mass to seed number *Y* = 0.02 + 0.0001*x*; *R* ^2^ = .32), which was then multiplied by the average inflorescence mass per plot to estimate seed output.

To assess the soil conditions in each plot, we took four soil samples, one at the midpoint of each transect in June 2012 and 2014. We used a 7.62 cm diameter auger to collect a 30 cm deep sample of mineral soil after measuring the depth of the organic horizon and removing it. Samples were assessed at Utah State University's (USU) Soil Analytics Laboratory for pH and electrical conductivity (Rhoades method), available phosphorus (Olsen NaHCO_3_ method), and organic matter (Walkley‐Black method; Pansu & Gautheyrou, 2007). Total nitrogen (TN) was assessed in 2012 soils by continuous‐flow direct combustion and mass spectrometry (CF‐IRMS) by the Stable Isotope Laboratory at USU. In 2014, we sampled nitrate‐N and ammonium‐N by placing soil subsamples into a 2 M KCl solution in the field, which were then shaken and filtered that day. We froze the extracts until they were processed on a Lachat flow injection auto‐analyzer (Lachat Chemicals). We calculated gravimetric soil moisture content for all years by measuring a 100–150 g subsample weight before and after it was dried in a drying oven for 24 hr at 105°C. We measured water depth and litter depth at every quadrat during vegetation sampling. To characterize the small differences in flood level in each plot, we collected and averaged four elevation points at the ends of the first and last transect in each plot using real‐time kinematic (RTK) satellite navigation.

### 2.5 Data analysis

We analyzed separately response variables of *Phragmites* cover; native, non‐invasive perennial cover; adjusted FQAI; species richness; and litter depth using linear mixed‐effect models (two models) with repeated measures in JMP version 13.1.0 (SAS Institute). Because the black plastic treatment was only monitored through 2014, we fitted two separate randomized, balanced, and incomplete block repeat measures ANOVA models for each response variable. The first statistical model included the fixed effects of treatment (CONT, SMBP, SGWM, SIWM, FGWM, and SMFG) and year (2013 and 2014). The second model included the fixed effects of treatment (CONT, SGWM, SIWM, FGWM, and SMFG; that is SMBP excluded), and year (2013, 2014, 2015, and 2016). All models included site, the interaction of site with treatment, and year as random factors. Pretreatment (2012) data are shown in figures but we excluded them from analyses because there was minimal variability among plots, and minimal correlation between pretreatment and post‐treatment values. To best meet the assumptions of normality and homogeneity of variance, we excluded the control treatment from the *Phragmites* and perennial cover models, transformed *Phragmites* and perennial percent cover using the logit of the proportion, and log transformed litter and species richness data. We analyzed log transformed inflorescence production (inflorescence/m^2^) using a linear mixed model ANOVA with repeated measures with treatment and year as fixed factors and site as a random factor. To assess seed viability, we conducted a mixed‐effects model with treatment as the fixed factor and site as the random factor. For analyses without evidence of interaction of treatment and year, we used Tukey post hoc means comparison tests. For analyses with significant interactions, we used contrasts for pertinent comparisons.

We performed two nonmetric multidimensional scaling (NMDS) ordinations using the package vegan (Oksanen et al., 2015) in R 3.3.1 (R Development Core Team, 2013). In the first ordination, we sought to visualize plant community trajectories over years 2014, 2015, and 2016 (i.e., when reference data were collected) in herbicide treatment plots relative to the untreated control and the target reference communities. In the second ordination, we sought to describe the influences of environmental variables on assembling plant communities, and the response of multiple plant guilds that are important to managers within the herbicide treated plots in 2016, the final monitoring year (Rohal et al., 2018). Plant guild metrics such as native perennials, graminoids, and forb cover were calculated by adding the cover of plants within each specific guild within a quadrat and then averaging quadrat cover values across plots. We chose to include multiple plant guilds, some of which contain species that overlap (e.g., native perennials that are also graminoids), in our ordination since these categories can have different importance, depending on management goals. To prepare for our ordinations, we excluded species that occurred in fewer than 5% of quadrats to reduce the disproportionate influence of rare species (McCune, Grace, & Urban, 2002). We calculated a dissimilarity matrix using Bray–Curtis and ran the ordinations using the R function metaMDS. We determined the appropriate number of dimensions by evaluating a scree‐plot, looking for the fewest dimensions that resulted in a plot with stress <20 (McCune et al., 2002). We correlated axis scores of both NMDS ordinations to guilds of the plant assemblages, environmental characteristics, and dominant species using Pearsons's correlation. We also conducted permutational multivariate analysis of variance (PERMANOVA) analyses in R package adonis using 999 permutations with Bray–Curtis distances to test for significant differences between plant communities between sites and years. PERMANOVA is a statistical test that compares the variability within groups with variability among different groups using a pseudo *F* statistic (Anderson, 2001). We first conducted a two‐way (with factors treatment and year) PERMANOVA, with sites as the strata within which to constrain ordinations using data as in the first ordination. We also ran one‐way PERMANOVAs with the 2016 data to determine the influence of site and treatment on plant community composition. We assessed Pearson's correlations between *Phragmites* cover, native perennial cover, and environmental metrics in the herbicide treated plots, analyzed together because we did not see significant differences in plant communities based on herbicide treatment (Miller, Belnap, Beatty, & Reynolds, 2006).

## 3 RESULTS

### 3.1 *Phragmites* cover

**Figure 2:**
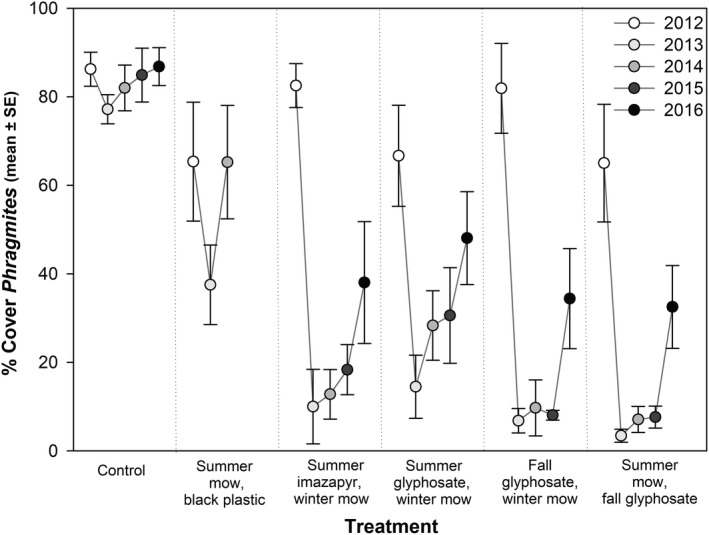
Cover of *Phragmites* following each treatment in each year. Pretreatment data were collected in June 2012, before initial treatments. Follow‐up treatments were conducted in 2013 and 2014

All herbicide treatments reduced *Phragmites* cover, but were not significantly different from one another (Table 1, Figure 2). *Phragmites* cover was lowest in 2013, following initial treatments, but increased over time across all herbicide treatments, with less variability in fall treated plots. The summer mow, black plastic treatment resulted in greater *Phragmites* cover than the herbicide treated plots in 2013 and 2014 (Table 1, Figure 2).

**Table 1:** Results of ANOVA tests for the effects of treatment, year, and their interaction on *Phragmites* cover

	*df*	*F*‐value	*p*‐value
Model 1: herbicide treatments, 2013–2016			
Year	3, 12	18.59	<.0001
Treatment	3, 12	1.69	.22
Year × Treatment	9, 35	0.56	.82
Model 2: herbicide treatments + black plastic, 2013–2014			
Year	1, 4	9.18	.04
Treatment	4, 16	6.61	.003
Year × Treatment	4, 16	1.28	.32

Note

Model 1 included all herbicide treatments (summer glyphosate winter mow, fall glyphosate winter mow, summer imazapyr winter mow, and summer mow fall glyphosate) for all post‐treatment years (2013–2016). Model 2 also included the black plastic treatment, but only included 2013–2014, the years black plastic was monitored.

### 3.2 *Phragmites* seed production

**Figure 3:**
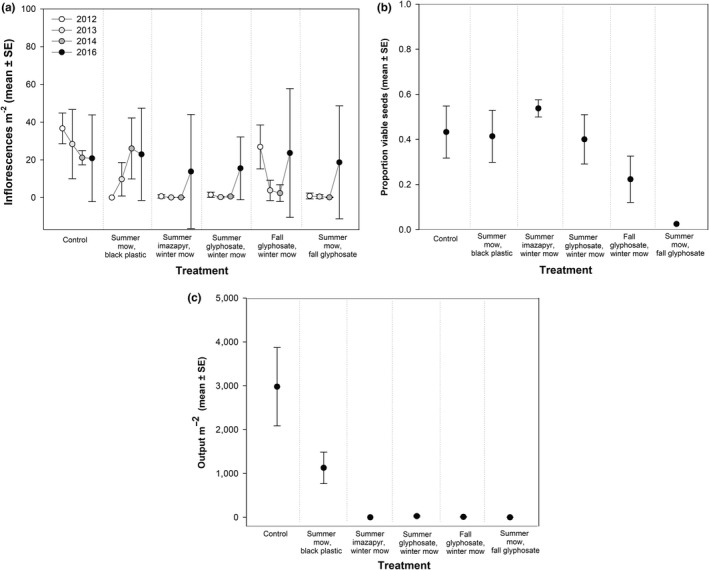
*Phragmites* inflorescence production, viability, and seed output following treatments. (a) Inflorescence production following each treatment. Data were collected in September each year, following fall herbicide applications. (b) The viability of *Phragmites* seeds. and (c) The output of *Phragmites* seeds in fall 2014, after the final follow‐up treatment

A significant treatment × year interaction was found for *Phragmites* inflorescence production (Table 2, Figure 3a). Following the initial 2012 treatments, all treatments with summer mowing or summer herbicide significantly reduced inflorescence numbers. The fall glyphosate, winter mow treatment did not significantly differ from the control (Table 2). In 2013 and 2014 follow‐up treatment years, all herbicide treatments had fewer inflorescences than the control. The viability of seeds in 2014 was not significantly different across treatments (*F*_(5,15)_ = 1.22, *p* = .34), though sample size was limited for summer imazapyr and summer mow, fall glyphosate due to limited inflorescence production in those treatments (Figure 3b). In 2014, output of seeds per meter squared was reduced by orders of magnitude between herbicide treated plots and the control (Figure 3c).

**Table 2:** Results of ANOVA tests for the effects of treatment, year, and their interaction on inflorescence production

	*df*	*F*‐value	*p*‐value
Year	3, 15	0.18	.91
Treatment	5, 19	24.40	<.0001
Year × Treatment	15, 57	13.47	<.001
Contrasts 2012, initial treatment year			
CONT versus SMBP	1, 73	90.80	<.0001
CONT versus SIWM	1, 73	26.71	<.0001
CONT versus SGWM	1, 73	22.54	<.0001
CONT versus FGWM	1, 73	0.004	.95
CONT versus SMFG	1, 73	31.33	<.0001
Contrasts 2013 + 2014, follow‐up treatment years			
CONT versus SMBP	1, 45	1.38	.25
CONT versus SIWM	1, 45	109.64	<.0001
CONT versus SGWM	1, 45	46.81	<.0001
CONT versus FGWM	1, 45	24.11	<.0001
CONT versus SMFG	1, 45	77.88	<.0001
Contrasts 2016, 2 years post‐treatments			
CONT versus SMBP	1, 73	0.12	.74
CONT versus SIWM	1, 73	2.75	.10
CONT versus SGWM	1, 73	0.62	.43
CONT versus FGWM	1, 73	0.40	.53
CONT versus SMFG	1, 73	1.04	.31

Abbreviations: CONT, Control; FGWM, fall glyphosate, winter mow; SGWM, summer glyphosate, winter mow; SIWM, summer imazapyr, winter mow; SMBP, summer mow, black plastic; SMFG, summer mow, fall glyphosate.

### 3.3 Native plant recovery and litter depth

**Figure 4:**
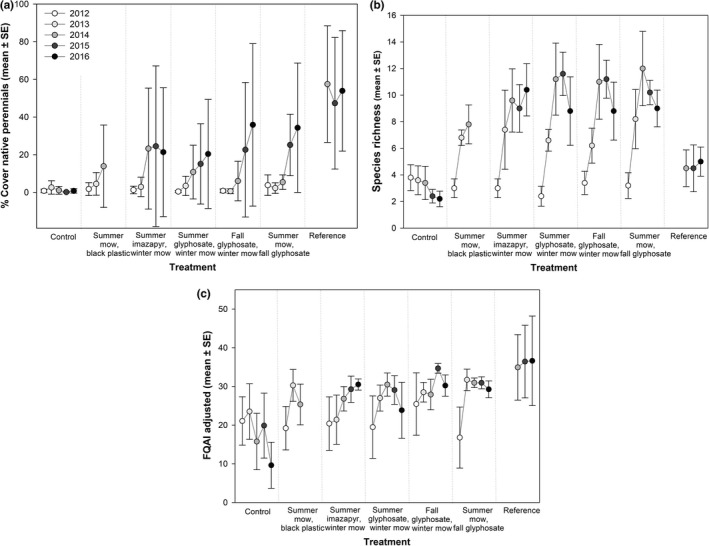
Plant community metrics following each *Phragmites* management treatment. (a) Cover of native perennials, (b) plot level species richness, and (c) adjusted Floristic Quality Assessment Index values following each treatment in management (2013–2014) and monitoring years (2015–2016). Pretreatment values are from 2012

Litter depth was highest in 2013 across all treatments and significantly lower in the summer mow, fall glyphosate treatment (Table S3, Figure S3). All herbicide treatments led to increases in native perennial plant cover, but they did not differ significantly (Table 3, Figure 4). Perennial plant cover was lowest in 2013, but increased significantly by 2015. Species richness did not differ significantly across herbicide treatments (Table 3, Figure 4), nor did the adjusted floristic quality assessment index (Table 3, Figure 4). The cover of native annuals was minimal across all treatments (Figure S4).

**Table 3:** Results of ANOVA tests for the effects of treatment, year, and their interaction on (a) native perennial cover, (b) species richness, and (c) adjusted Floristic Quality Assessment Indices

	*df*	*F*‐value	*p*‐value
(a) Native perennial cover			
Model 1: herbicide treatments, 2013–2016			
Year	3, 15	6.32	.005
Treatment	3, 12	0.78	.53
Year × Treatment	9, 34	1.93	.08
Model 2: herbicide treatments + black plastic, 2013–2014			
Year	1, 5	11.91	.02
Treatment	4, 15	1.54	.24
Year × Treatment	4, 16	0.91	.48
(b) Species richness			
Model 1: herbicide treatments + control, 2013–2016			
Year	3, 15	1.69	.21
Treatment	4, 15	22.12	<.0001
Year × Treatment	12, 46	1.76	.08
Model 2: all treatments, 2013–2014			
Year	1, 5	2.5	.17
Treatment	5, 19	8.7	.0002
Year × Treatment	5, 19	1.03	.43
(c) Adjusted FQAI			
Model 1: herbicide treatments + control, 2013–2016			
Year	3, 6	3.03	.12
Treatment	4, 15	4.5	.01
Year × Treatment	12, 52	1.13	.36
Model 2: all treatments, 2013–2014			
Year	1, 4	0.23	.65
Treatment	5, 19	2.12	.11
Year × Treatment	5, 21	0.89	.50

**Figure 5:**
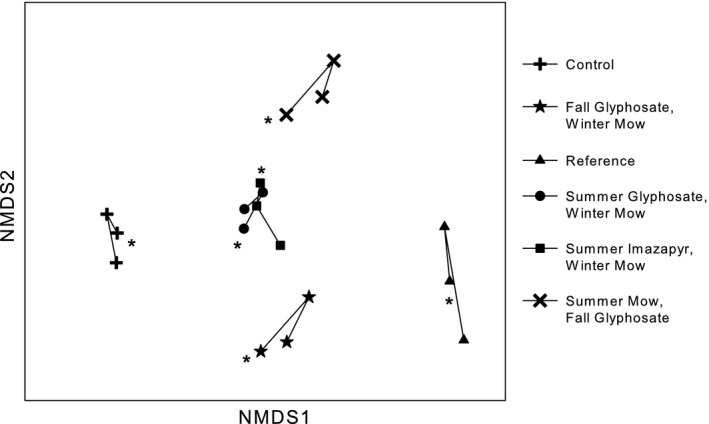
Multidimensional scaling plot of plant community assemblage centroids in years 2014–2016. Asterisks are adjacent to the 2016 centroids, the final year of data collection. Stress = 13.8

Plant community composition in herbicide treated plots was significantly different than both the control and the reference plots (Figure 5, PERMANOVA *F*_(5,87)_ = 7.02, *p* = .001, *R* ^2^ = .29). Plant communities in 2014 were significantly different than they were in 2016 (Figure 5, PERMANOVA *F*_(2,90)_ = 1.84, *p* = .01, *R* ^2^ = .03). NMDS Axis 1 represented a gradient from *Phragmites*‐dominated communities to native perennial communities dominated by graminoids (Figure 5, Table 4). NMDS Axis 2 represented a gradient from native bulrushes to annuals (Figure 5, Table 4), while Axis 3 was driven by water depth (Table 4).

**Table 4:** Pearson's correlations between NMDS axis scores and dominant species' covers, vegetation guild covers, and environmental metrics from the ordination of control, reference, and herbicide treatment plots in 2014–2016

	NMDS1	NMDS2	NMDS3
Dominant species			
*Phragmties australis*	**−0.84**	−0.1	0.03
*Bolboshoenus maritimus*	**0.38**	0.05	0.35
*Distichlis spicata*	**0.56**	−0.18	−0.15
*Typha* spp.	0	**0.45**	0.12
*Schoenoplectus americanus*	0.18	**0.66**	−0.2
*Berula erecta*	−0.01	**0.46**	−0.19
Vegetation guilds			
Graminoids	**0.67**	0.1	−0.4
Forbs	0.12	**0.3**	**−0.21**
Native annuals	**0.2**	**−0.33**	−0.11
Native perennials	**0.57**	**0.38**	−0.3
Bulrushes	**0.34**	**0.57**	−0.03
Environmental metrics			
Litter depth	**−0.33**	**0.31**	−0.09
Water depth	0.1	0.07	**0.64**

Note

Bolded values are statistically significant (*p* ≤ .05).

### 3.4 Environmental influences on assembling plant communities

**Figure 6:**
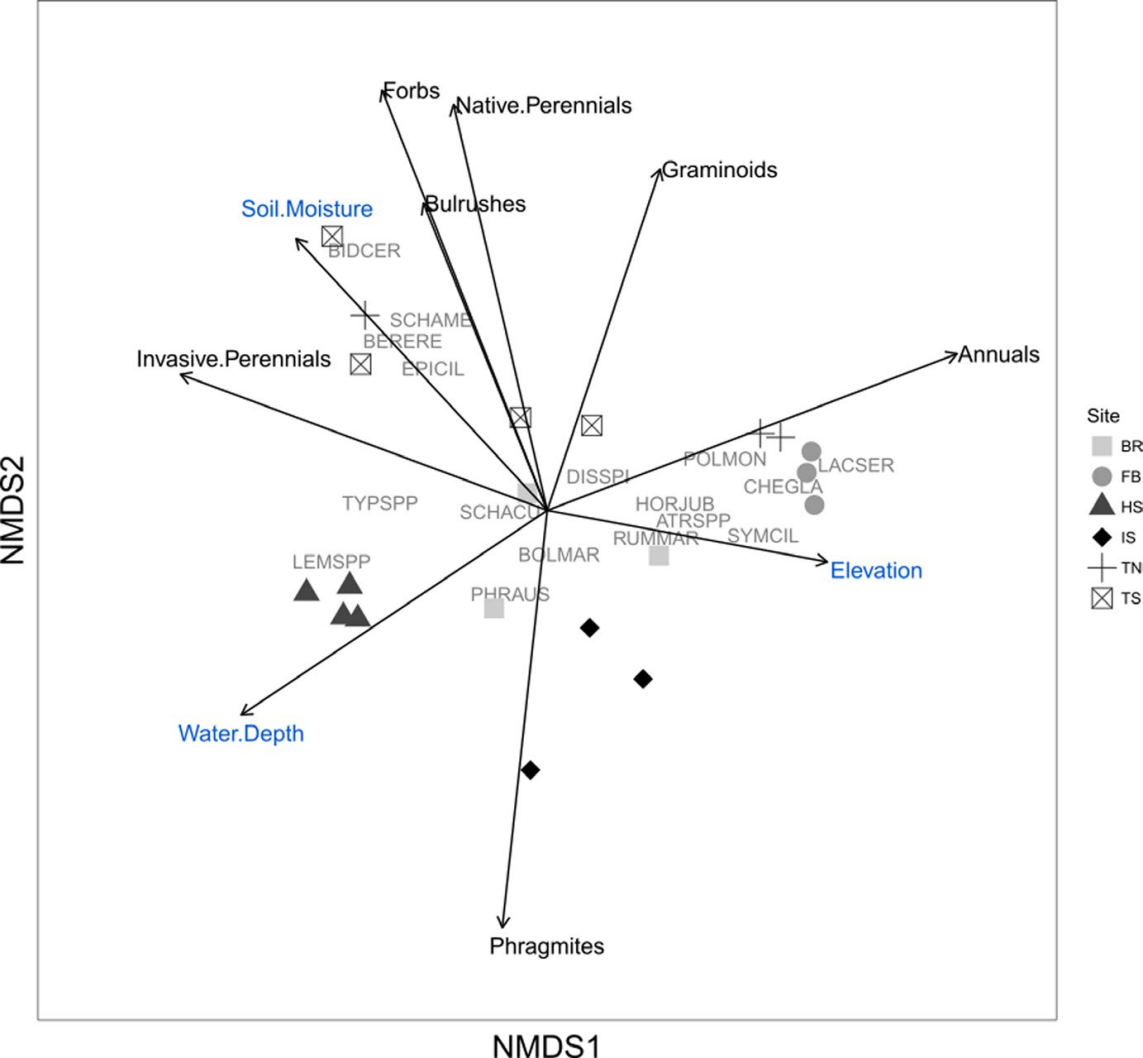
Multidimensional scaling biplot of herbicide treated plots (FGWM, SGWM, SIWM, and SMFG) in 2016 with plot scores coded by site. Environmental variables (blue) and species guilds (black) overlaid are restricted to those variables that had >0.55 correlation with NMDS axes (Table 5). Species codes are ATRSPP: *Atriplex* spp., BERERE: *Berula erecta*, BOLMAR: *Bolboschoenus maritimus*, BIDCER: *Bidens cernua*, CHEGLA: *Chenopodium glaucum*, DISSPI: *Distichlis spicata*, EPICIL: *Epilobium ciliatum*, HORJUB: *Hordeum jubatum*, LACSER: *Lactuca serriola*, LEMSPP: *Lemna* spp., PHRAUS: *Phragmites australis*, POLMON: *Polypogon monspeliensis*, RUMMAR: *Rumex maritimus*, SCHACU: *Schoenoplectus acutus*, SCHAME: *Schoenoplectus americanus*, SYMCIL: *Symphyotrichum ciliatum*, TYPSPP: *Typha* spp. See Figure 1 for site codes. Stress = 8.54

**Figure 7:**
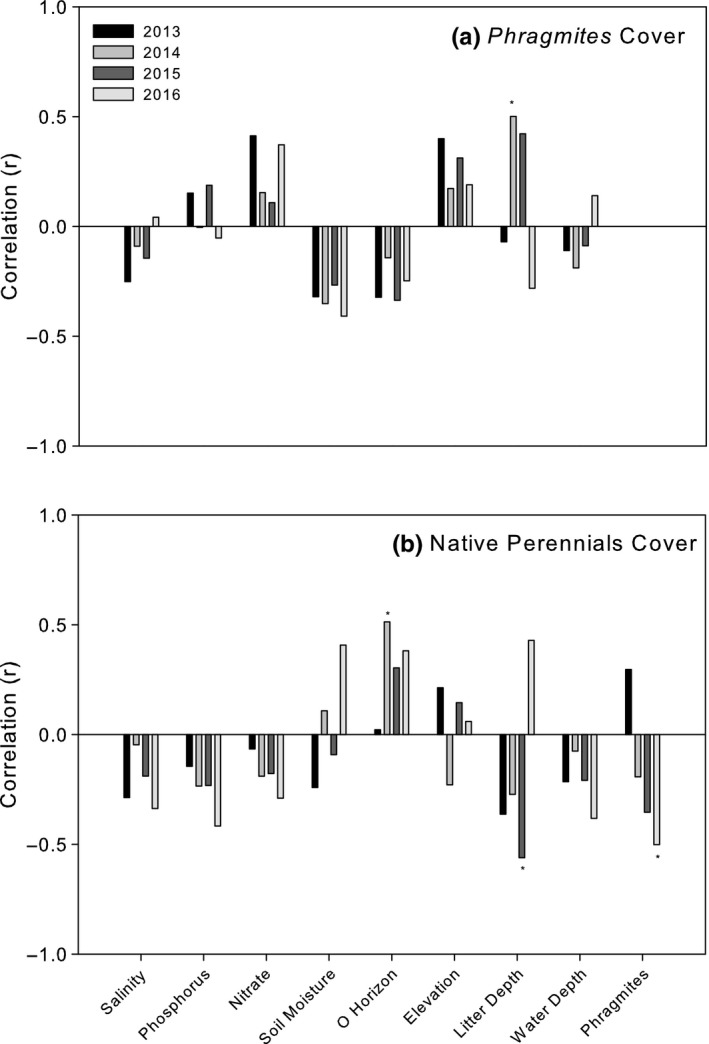
Pearson's correlations between (a) *Phragmites* cover and environmental metrics and (b) perennial cover and environmental metrics, in grouped herbicide treated plots. Significant correlations (*p* ≤ .05) are marked with an asterisk

We evaluated the influence of site, treatment, and environmental factors on plant communities from the herbicide treated plots in 2016. Communities did not differ significantly by treatment (PERMANOVA *F*_(3,16)_ = 0.56, *p* = .64, *R* ^2^ = .09), but were different by site (PERMANOVA *F*_(5,14)_ = 3.84, *p* = .001, *R* ^2^ = .58), and showed separation by site in the NMDS ordination (Figure 6). NMDS Axis 1 primarily represented a hydrologic gradient, from higher moisture to dry, which was reflected by a gradient of obligate emergent plants to opportunist annuals (Table 5). Axis 2 represented a gradient from *Phragmites*‐dominated communities to communities dominated by native perennials (Table 5). Soil moisture and o‐horizon depth were positively correlated with this axis, which indicates an association between wetter plots and greater native perennial cover and less *Phragmites* cover. This finding was reflected in the consistent trend of negative Pearson's correlations between *Phragmites* cover and soil moisture (2013: *r* = −.32, *p* = .17; 2014: *r* = −.35, *p* = .12; 2015: *r* = −.27, *p* = .25; and 2016: *r* = −.41, *p* = .07) and o‐horizon depth (2013: *r* = −.32, *p* = .17; 2014: *r* = −.14, *p* = .54; 2015: *r* = −.34, *p* = .26; and 2016: *r* = −.25, *p* = .29), and the positive relationship between *Phragmites* cover and elevation over time (2013: *r* = .4, *p* = .08; 2014: *r* = .17, *p* = .46; 2015: *r* = .31, *p* = .18; and 2016: *r* = .19, *p* = .42; Figure 7). Pearson correlations between native perennials and environmental metrics showed a consistent negative relationship between perennials and phosphorus (2013: *r* = −.14, *p* = .54; 2014: *r* = −.23, *p* = .31; 2015: *r* = −.23, *p* = .32; and 2016: *r* = −.42, *p* = .07), nitrate (2013: *r* = −.07, *p* = .78; 2014: *r* = −.19, *p* = .42; 2015: *r* = −.18, *p* = .45; and 2016: *r* = −.29, *p* = .21), water depth (2013: *r* = −.21, *p* = .36; 2014: *r* = −.08, *p* = .75; 2015: *r* = .21, *p* = .37; and 2016: *r* = −.38, *p* = .09), and a consistent positive relationship with o‐horizon depth (2013: *r* = .02, *p* = .93; 2014: *r* = .51, *p* = .02; 2015: *r* = .3, *p* = .19; and 2016: *r* = .38, *p* = .09; Figure 7).

**Table 5:** Pearson correlation coefficients between NMDS axis scores and dominant species, vegetation guilds, and environmental metrics from the ordination of herbicide treated plots in 2016, the final monitoring year

	NMDS1	NMDS2	NMDS3
Dominant species			
*Phragmites australis*	−0.12	**−0.81**	−0.15
*Typha* spp.	**−0.73**	0.19	−0.09
*Lemna* spp.	**−0.54**	−0.3	0.04
*Distichlis spicata*	0.06	0.07	**−0.49**
*Schoenoplectus acutus*	0	0.02	**−0.64**
*Bolboshoenus maritimus*	−0.01	−0.05	**−0.62**
*Schoenoplectus americanus*	−0.35	**0.61**	0.24
*Berula erecta*	−0.34	**0.54**	−0.04
Vegetation guilds			
Bulrushes	−0.31	**0.56**	−0.12
Graminoids	0.29	**0.63**	−0.04
Forbs	−0.42	**0.77**	0.03
Introduced annuals	**0.82**	0.23	−0.04
Native annuals	**0.62**	0.17	−0.09
Invasive perennials	**−0.73**	0.2	−0.09
Native perennials	−0.25	**0.77**	−0.04
Environmental metrics			
Phosphorus	0.17	−0.36	**0.55**
Soil moisture	**−0.57**	**0.44**	−0.19
Water depth	**−0.64**	−0.31	0.07
Litter depth	−0.28	**0.46**	0.22
Elevation	**0.55**	−0.07	−0.18
O‐Horizon depth	**−0.54**	**0.48**	0.01

Note

Bolded values are statistically significant (*p* ≤ .05).

## 4 DISCUSSION

Though *Phragmites* is one of the most studied plant invaders in North America (Meyerson et al., 2016) and seen as a model species for understanding what makes invasions successful (Eller et al., 2017), *Phragmites* management is comparatively understudied, while past studies are limited by short time frames and small plot sizes (Hazelton et al., 2014; Quirion, Simek, Dávalos, & Blossey, 2017). In this multi‐site, large‐plot, 5‐year study, we found that contrary to our expectations, fall applications were more consistently effective at reducing *Phragmites* cover across sites and years, while initially treating *Phragmites* in summer with mowing or herbicide can greatly reduce its propagule pressure. Native plant recruitment was minimal in the first 2 years after all herbicide treatments, likely because of the dense *Phragmites* litter layer after mowing. Restoration success was context dependent—sites with higher levels of soil moisture resulted in plant communities with less *Phragmites* cover and more native perennials. Site hydrology played an important role in treatment effectiveness and early plant succession, particularly in this semiarid system where water for wetlands can be in short supply (Downard et al., 2014). Thus, water availability should be considered in *Phragmites* management planning, a lesson likely applicable to wetland invasive management in other semiarid regions.

### 4.1 *Phragmites* management outcomes are more variable following summer herbicide applications

Contrary to our expectations and prior research in shorter‐term studies (Derr, 2008; Mozdzer et al., 2008), with additional years of applications and longer‐term monitoring, we found that fall applications resulted in consistently low *Phragmites* cover across environmentally variable sites and a slower return of *Phragmites* over time. In contrast, despite repeated follow‐up treatments in the summer herbicide plots, we observed an increasing cover of *Phragmites* during treatment years. This finding supports herbicide label recommendations for application in the fall, when absorbed herbicides can be translocated along with the carbohydrates *Phragmites* sends to rhizomes in preparation for senescence (Tu et al., 2001). Also, we found no significant advantage to imazapyr over glyphosate using summer applications in contrast to Derr (2008) and Mozdzer et al. (2008). Thus, the benefits of imazapyr may not justify its increased cost. Invasive plants frequently reinvade management areas (Petrov & Marrs, 2000), because they often take advantage of the high resource availability associated with the physical disturbances that occur with management (Davis, Grime, Thompson, Davis, & Philip, 2000). Our results highlight the need to continue follow‐up management efforts beyond the typical 3‐year herbicide sequence to ensure *Phragmites* remains at a low cover, particularly where native plants (which can delay or prevent *Phragmites* reinvasion) are slow to return. The observed trend of increasing *Phragmites* cover after herbicide treatments ceased is in line with the growing consensus that without continuing treatment of the remaining *Phragmites*, reinvasion is likely (Hazelton et al., 2014; Kettenring & Adams, 2011).

### 4.2 Treatments differentially influence *Phragmites* inflorescence production

Given the recent understanding that *Phragmites* uses seed dispersal as a primary means for spread (Kettenring et al., 2011; Kettenring & Mock, 2012), the reinvasion by *Phragmites* following management is likely influenced by seed‐based recruitment. *Phragmites* recruitment success increases with propagule pressure (Byun, de Blois, & Brisson, 2015), which makes reducing seed availability important for limiting *Phragmites* reinvasion. In line with our hypothesis, this study demonstrated that there are multiple ways to limit *Phragmites* inflorescence production—using both summer herbicide applications and summer mowing—but only mowing in combination with a fall glyphosate spray also had consistent multi‐year reduction of *Phragmites* cover. In follow‐up treatment years, all herbicide treatments had very little inflorescence production, perhaps because the remaining *Phragmites* was too stressed from the previous years of management, or represented new recruits that did not produce inflorescences in the establishment year. Thus, it is most critical to mow *Phragmites* in the summer before the initial treatment year to reduce inflorescence production. Unfortunately, summer mowing has many logistical challenges including the difficulty of getting marsh machinery into wetlands during wetter periods and the potential to disrupt bird nesting, which is prohibited by U.S. Federal law (i.e., the Migratory Bird Treaty Act of 1918, 16 U.S.C. §§ 703–712). High‐intensity summer livestock grazing also has the potential to reduce *Phragmites* inflorescence production (Duncan, 2019; Duncan et al., 2019; Silliman et al., 2014) and could be used in replacement of mowing in some management sequences. We observed *Phragmites* inflorescence production increase once treatments ceased, as *Phragmites* began to reinvade, further emphasizing the importance of continuing follow‐up treatments beyond a 3‐year cycle. *Phragmites* can also reproduce and spread via rhizomes and stolons, so care should be taken to minimize the transportation of these vegetative propagules during management activities.

### 4.3 Native plant recovery outcomes vary by site, not treatment

While invasive plant removal treatments are often selected based on the best method for reducing cover of the invader, treatments can have differential impacts on the response of the native plant community (Flory, 2010; Mason & French, 2007). Surprisingly, imazapyr did not restrict native plant recruitment any more than glyphosate (despite differing modes of action and half‐lives), perhaps because its persistence in the anaerobic conditions associated with moist wetland soil is far lower than aerobic conditions (Wang, Wang, & Fan, 2006). Differences in native plant recovery due to the timing of herbicide application were also not discernable, likely because of large variability in native plant recovery across sites. Instead, initial native plant recruitment was likely low because of the deep litter layer that remained after mowing *Phragmites*, which shaded the soil surface and prevented the germination of native seeds (Kettenring, 2016). Shallowly flooded *Phragmites* litter degrades quickest (Voellm & Tanneberger, 2014), which more quickly provides light for seed germination. Managers of wetlands with water control structures can flood sites following mowing to facilitate litter decomposition (Rohal, Hambrecht, Cranney, & Kettenring, 2017).

Recruitment deficiencies may also be partly due to propagule limitation, which has led to poor native plant recruitment in many ecosystem restorations (French, Mason, & Sullivan, 2011; Kettenring & Galatowitsch, 2011). While some studies in tidal ecosystems have found diverse native seed banks under *Phragmites* (e.g., Hazelton, Downard, Kettenring, McCormick, & Whigham, 2018), the composition and densities may not match noninvaded areas and may be highly variable across sites. The reference wetlands in this study were dominated by three bulrush species that are important habitat and provide energy‐rich seed forage for migratory birds (Downard et al., 2017), an ecosystem function that is a main goal of local restoration efforts (Rohal et al., 2018). The assembling plant communities in our treatment plots, however, had low cover of bulrush species (<10% cover in most plots), which indicates that this goal may not be met without further restoration action.

### 4.4 Environmental variability determines management success and native plant recovery

The ecological contingencies of treatment effectiveness for invasive removal and native plant recovery are poorly understood (Flory, 2010; Kettenring & Adams, 2011; Rohal, Cranney, & Kettenring, 2019). Contrary to our expectations, we did not observe a significant influence of nutrients on *Phragmites* cover. While excess nutrients favor *Phragmites*, a nutrient specialist, in competitive dynamics between returning *Phragmites* and native species (Kettenring et al., 2011; Mozdzer & Zieman, 2010), our results indicate that hydrology had the more dominant influence on plant community outcomes, which likely obscured the role of nutrients. Specifically, *Phragmites* cover was more effectively reduced, and stayed at a lower cover after treatments ceased, in lower compared with higher elevation sites; low elevation sites had higher soil moisture and deeper o‐horizons, indications of sustained flooding throughout the growing season (Reddy & DeLaune, 2008). Consistent with our hypothesis, the driest sites saw inadequate cover reduction of *Phragmites* throughout the course of the experiment, likely because herbicide uptake is disrupted when plants are stressed (Tu et al., 2001). Contrary to our expectations, summer herbicide applications were not more effective than fall applications at *Phragmites* removal in drought stressed locations, as *Phragmites* cover remained high following all treatments in drought prone areas. Therefore, spraying herbicides should be avoided at relatively high elevation and dry sites where *Phragmites* is subjected to water stress. These findings suggest drought represents an important restoration threshold, a point at which the likely outcome of restoration does not justify the costs. Herbicide‐based restoration is thus likely to be constrained in arid wetlands where water scarcity is an ever‐increasing issue (Meyerson et al., 2010; Rohal et al., 2019).

Native perennial recruitment was also higher at sites with high soil moisture content, likely because *Phragmites* was more effectively removed, which opened‐up limiting resources, and because these conditions favored the establishment of wetland species. This higher native plant recruitment also likely limited the reinvasion of *Phragmites*, particularly by seed (Byun et al., 2015). Counterintuitively, higher *Phragmites* cover and lower native perennial cover were associated with deeper water, common in areas with artificial hydrologic control. Deeper water likely restricted germination and establishment of native species, and the reinvasion of *Phragmites* by seed (Galatowitsch, Larson, & Larson, 2016), but still allowed for clonal expansion of remnant *Phragmites* rhizomes (Amsberry, Baker, Ewanchuk, & Bertness, 2000). These findings are consistent with Carlson, Kowalski, and Wilcox (2009), who found a significant influence of topography on *Phragmites* cover following management, with higher cover of *Phragmites* in higher elevations and limited native plant recovery in deeper water. Sites with higher soil moisture and sustained flooding throughout the growing season (conditions found in less disturbed sites with more natural hydrologic patterns) are likely to have better *Phragmites* cover reduction, but where the water depth is deep, are unlikely to see robust native plant recovery to compete with reinvading *Phragmites*. Managers could use these hydrologic contingencies to map priority management areas that are likely to have *Phragmites* removal success (such as areas with impoundments for water control, or areas known to have persistent soil moisture) and to map expectations for native plant recolonization outcomes and possible revegetation needs (e.g., Long, Kettenring, & Toth, 2017).

## 5 CONCLUSION

Applied scientists increasingly recognize that restoration outcomes are highly influenced by uncontrolled spatial and temporal contingencies in addition to management decisions, though this concept has been infrequently applied to invasive species driven restorations (Grman, Bassett, & Brudvig, 2013; Rohal et al., 2019). This acknowledgment has led to a call to compare restoration outcomes from similar approaches across sites and to interpret the variability to improve prediction in restoration and inform restoration planning (Brudvig et al., 2017). Given limited resources and the broad geographic scope of the invasion, *Phragmites* managers must often choose to prioritize some patches for management, while taking a hands‐off approach at other locations. Knowing the environmental circumstances that promote restoration success can help inform this decision‐making and allow for the most cost‐effective management. Our multi‐site study found wide variability in *Phragmites* management outcomes and native plant recovery, which indicates success in the restoration of *Phragmites*‐invaded wetlands is highly context dependent, a finding consistent with studies in other regions of North America (Carlson et al., 2009; Hazelton, 2018; Quirion et al., 2017). We found that site hydrology played an important role in determining outcomes, but there were likely other unmeasured factors that contributed to divergent results such as landscape setting, site history, and age of invasion, which should be further explored. A more detailed examination of how differing temporal patterns in hydrology influence *Phragmites* cover reduction and native plant outcomes is also warranted. Despite high variability across sites, the summer mowing with fall glyphosate‐spraying treatment had the best outcome in terms of reducing *Phragmites* cover, inflorescence number, and litter depth. The inconsistent results we found in the cover and quality of native plants following all treatments highlight the need to incorporate revegetation with *Phragmites* management in future research and management efforts (Byun et al., 2015; Hazelton et al., 2014; Rohal et al., 2017). The variability we observed emphasizes the importance of replicating invasive species management experiments across many sites so conclusions will not be skewed by uniquely favorable or unfavorable conditions, and the context of successes and failures can be understood.

## CONFLICT OF INTEREST

We have no conflict of interests.

## AUTHORS CONTRIBUTIONS

KK, EH, CC, and CR designed methodology and implemented treatments; CC and CR collected data; CR analyzed data; CR and KK led the writing of the manuscript. All authors contributed critically to the drafts and gave approval for publication.

## Data Availability

Data files supporting the results are archived in Figshare (https://doi.org/10.6084/m9.figshare.10007237.v1).
