# Supplementary material for: Invasive Phragmites australis management outcomes and native plant recovery are context dependent
Source: Ecol Evol. 2019 Dec 5;9(24):13835–49. doi: 10.1002/ece3.5820 (PMC6953697; doi:10.1002/ece3.5820)
Supplement: Supplementary file 1 [file ECE3-9-13835-s001.docx]

**Supplementary Material**

**Invasive *Phragmites australis* Management Outcomes and Native Plant Recovery are Context Dependent**

**Christine B. Rohal, Chad Cranney, Eric L.G. Hazelton, and Karin M. Kettenring**

Table S1. Environmental data averaged by site (± 1 standard error). Environmental variables are pH, salinity (dS/m), organic matter (%), Total Nitrogen (mg/kg), Nitrate (mg/kg), Ammonium (mg/kg), Phosphorus (mg/kg), organic horizon depth (cm), elevation (m above sea level), and soil moisture (percent dry weight, averaged across all monitoring years). All data was collected in 2012 before treatments were enacted except N03- and NH4, which were collected in 2014. Sites are also characterized by their expected conditions regarding hydrology, nutrient enrichment, and salinity.

Sites are BR: Bear River National Wildlife Refuge, FB: Farmington Bay Waterfowl Management Area, HS: Howard Slough Waterfowl Management Area, IS: Inland Sea Shorebird Reserve: TN: The Nature Conservancy Shorelands Preserve – North, TS: The Nature Conservancy Shorelands Preserve – South.

|  | **BR** | **FB** | **HS** | **IS** | **TN** | **TS** |
| --- | --- | --- | --- | --- | --- | --- |
| pH | 7.5±0.06 | 7.8±0.06 | 7.8±0.04 | 8.0±0.02 | 7.9±0.04 | 7.9±0.02 |
| Salinity | 9.5±0.52 | 11.2±2.21 | 20.4±2.35 | 6.4±0.91 | 4.2±0.57 | 6.6±0.90 |
| Organic | 2.5±0.12 | 3.9±0.38 | 4.1±0.30 | 2.7±0.11 | 3.0±0.25 | 3.0±0.19 |
| TN | 1387.2±180 | 2949.4±444 | 2482.4±196 | 1144.9±52 | 1126.8±64 | 1171.5±107 |
| N03- | 0±0 | 0.02±0.01 | 0±0 | 0.92±0.17 | 0.14±0.04 | 0±0 |
| NH4 | 3.4±0.84 | 121.0±36.4 | 32.3±8.39 | 3.2±0.69 | 3.3±0.74 | 2.3±0.26 |
| P | 21.1±1.6 | 68.2±7.9 | 77.7±9.2 | 70.1±0.9 | 31.1±2.6 | 28.2±1.4 |
| O Horizon | 3.9±0.5 | 2.1±0.48 | 7.2±0.68 | 2.15±0.25 | 7.0±0.82 | 8.5±0.63 |
| Elevation | 1282.9±0.02 | 1282.6±0.04 | 1281.7±0.03 | 1283.3±0.02 | 1282.9±0.07 | 1282.3±0.05 |
| Moisture | 53.4±4.9 | 77.5±5.8 | 80.0±3.3 | 36.7±6.1 | 53.6±4.2 | 62.0±4.4 |
|  |  |  |  |  |  |  |
| Hydrology | Drought prone | Impounded wetland | Impounded wetland | Drought prone | Unaltered hydrology | Unaltered hydrology |
| Nutrient enrichment | Non-eutrophic | Eutrophic | Eutrophic | Eutrophic | Non-eutrophic | Non-eutrophic |
| Salinity | Medium | Medium | High | High | Low | Low |

Table S2. Common plant species of Great Salt Lake wetlands and their assigned C values (Coefficient of Conservatism values) for Utah wetlands. Wetland indicator status abbreviations are OBL: Obligate, FAC: Facultative, FACW: Facultative Wetland, and FACU: Facultative Upland.

| Species name | Family | Duration | Growth Habit | Nativity | Wetland Indicator Status^1^ | C value^2^ |
| --- | --- | --- | --- | --- | --- | --- |
| *Atriplex prostrata* | Chenopodiaceae | Annual | Forb/herb | Introduced | FACW | 0 |
| *Bassia scoparia* | Chenopodiaceae | Annual | Forb/herb | Introduced | FAC | 0 |
| *Berula erecta* | Apiaceae | Perennial | Forb/herb | Native | OBL | 6 |
| *Bidens cernua* | Asteraceae | Annual | Forb/herb | Native | OBL | 4 |
| *Cardaria draba* | Brassicaceae | Perennial | Forb/herb | Introduced |  | 0 |
| *Chenopodium rubrum* | Chenopodiaceae | Annual | Forb/herb | Native | FACW | 3 |
| *Distichlis spicata* | Poaceae | Perennial | Graminoid | Native | FAC | 4 |
| *Eleocharis palustris* | Cyperaceae | Perennial | Graminoid | Native | OBL | 4 |
| *Epilobium ciliatum* | Onagraceae | Perennial | Forb/herb | Native | FACW | 3 |
| *Hordeum jubatum* | Poaceae | Perennial | Graminoid | Native | FAC | 2 |
| *Hordeum marinum* | Poaceae | Annual | Graminoid | Introduced | FAC | 0 |
| *Juncus arcticus* | Juncaceae | Perennial | Graminoid | Native | FACW | 3 |
| *Lactuca serriola* | Asteraceae | Annual, Biennial | Forb/herb | Introduced | FACU | 0 |
| *Lemna minor* | Lemnaceae | Perennial | Forb/herb | Native | OBL | 2 |
| *Lythrum salicaria* | Lythraceae | Perennial | Subshrub, Forb/herb | Introduced | OBL | 0 |
| *Mimulus guttatus* | Scrophulariaceae | Annual, Perennial | Forb/herb | Native | OBL | 5 |
| *Polygonum lapathifolium* | Polygonaceae | Annual | Forb/herb | Native | FACW | 1 |
| *Polygonum ramosissimum* | Polygonaceae | Annual | Forb/herb | Native | FAC | 2 |
| *Polypogon monspeliensis* | Poaceae | Annual | Graminoid | Introduced | FACW | 0 |
| *Puccinellia distans* | Poaceae | Perennial | Graminoid | Introduced | FACW | 0 |
| *Ranunculus cymbalaria* | Ranunculaceae | Perennial | Forb/herb | Native | OBL | 4 |
| *Ranunculus sceleratus* | Ranunculaceae | Annual, Perennial | Forb/herb | Native | OBL | 3 |
| *Rorippa palustris* | Brassicaceae | Annual, Biennial, Perennial | Forb/herb | Native | OBL | 4 |
| *Rumex maritimus* | Polygonaceae | Annual, Biennial | Forb/herb | Native | FACW | 3 |
| *Sagittaria cuneata* | Alismataceae | Perennial | Forb/herb | Native | OBL | 7 |
| *Salicornia rubra* | Chenopodiaceae | Annual | Subshrub, Forb/herb | Native | OBL | 5 |
| *Schoenoplectus acutus* | Cyperaceae | Perennial | Graminoid | Native | OBL | 5 |
| *Schoenoplectus americanus* | Cyperaceae | Perennial | Graminoid | Native | OBL | 4 |
| *Schoenoplectus maritimus* | Cyperaceae | Perennial | Graminoid | Native | OBL | 6 |
| *Sonchus asper* | Asteraceae | Annual | Forb/herb | Introduced | FAC | 0 |
| *Spergularia salina* | Caryophyllaceae | Annual, Biennial, Perennial | Forb/herb | Native | OBL | 4 |
| *Suaeda calceoliformis* | Chenopodiaceae | Annual, Perennial | Forb/herb | Native | FACW | 3 |
| *Typha domingensis* | Typhaceae |  |  | Native | OBL | 3 |
| *Typha latifolia* | Typhaceae | Perennial | Forb/herb | Native | OBL | 2 |
| *Veronica anagallis-aquatica* | Scrophulariaceae | Biennial, Perennial | Forb/herb | Native | OBL | 1 |
| *^1^ USDA, NRCS. 2019. The PLANTS Database* ([http://plants.usda.gov](http://plants.usda.gov/), 6 September 2019). National Plant Data Team, Greensboro, NC 27401-4901 USA.  *^2^* Menuz, D., Sempler, R., & Jones, J. 2016. Assessment of wetland condition and wetland mapping accuracy in Upper Blacks Fork and Smiths Fork, Uinta Mountains, Utah. Utah Geological Survey, Salt Lake City, UT. | | | | | | |
|  | | | | | | |
|  | | | | | | |

Table S3. Results of ANOVA tests for the effects of treatment, year, and their interaction on litter depth. Treatment codes are CONT: Control, SGWM: Summer Glyphosate, Winter Mow, SIWM: Summer Imazapyr, Winter Mow, FGWM: Fall Glyphosate, Winter Mow, SMFG: Summer Mow, Fall Glyphosate.

| **Model 1: Herbicide treatments + control, 2013-2016** | **DF** | **F-value** | **P-value** |
| --- | --- | --- | --- |
| Year | 3, 15 | 1.72 | 0.2045 |
| Treatment | 4, 15 | 26.04 | 0.001 |
| Year*Treatment | 12, 46 | 4.41 | 0.001 |
| **Contrasts, 2013-2016** |  |  |  |
| CONT vs SGWM | 1,15 | 17.44 | 0.0008 |
| CONT vs SIWM | 1,15 | 19.50 | 0.0005 |
| CONT vs FGWM | 1,15 | 15.57 | 0.0013 |
| CONT vs SMFG | 1,15 | 102.12 | <0.0001 |
| SMFG vs SGWM | 1,15 | 35.17 | <0.0001 |
| SMFG vs SIWM | 1,15 | 32.37 | <0.0001 |
| SMFG vs FGWM | 1,15 | 37.94 | <0.0001 |
| SGWM vs SIWM | 1,15 | 0.06 | 0.81 |
| SGWM vs FGWM | 1,15 | 0.05 | 0.82 |
| FGWM vs SIWM | 1,15 | 0.22 | 0.65 |
|  |  |  |  |
| **Model 2: All treatments, 2013-2014** | | | |
| Year | 1, 4 | 36.35 | 0.003 |
| Treatment | 5, 19 | 5.46 | 0.003 |
| Year*Treatment | 5, 22 | 2.32 | 0.08 |

**
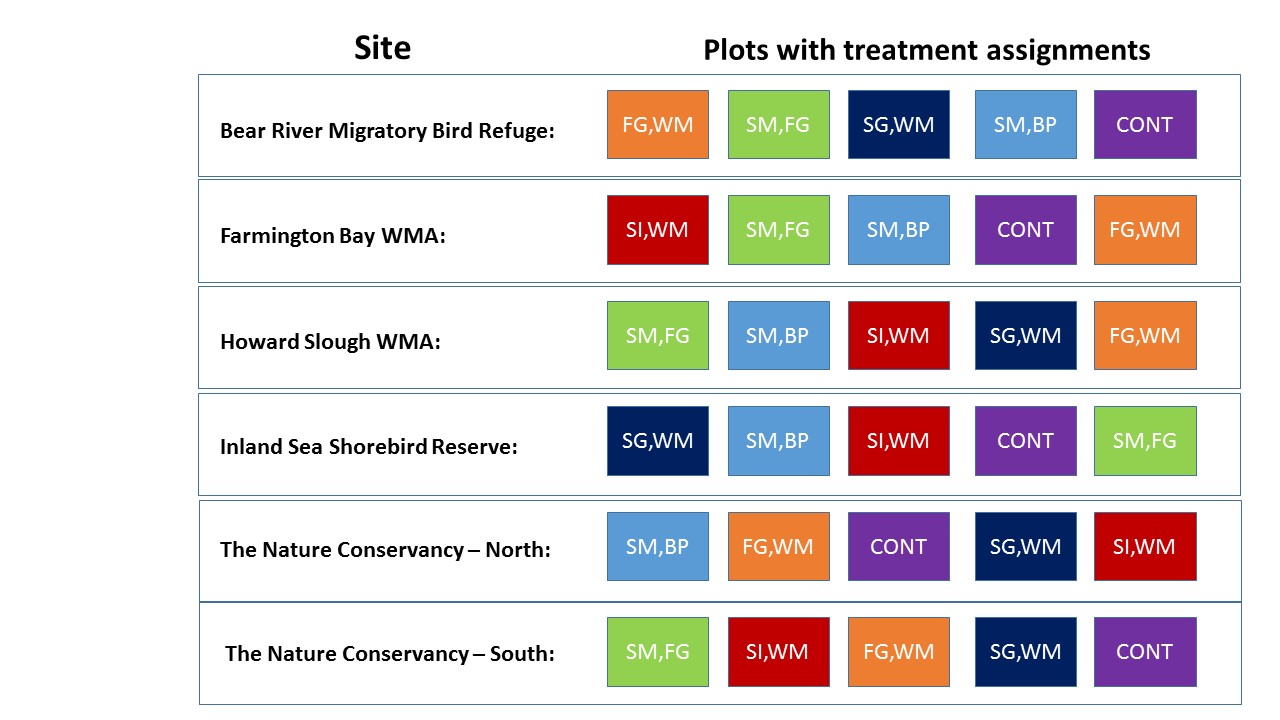
**

Figure S1. Visualization of overall experimental design – a randomized, balanced, incomplete block design. *Phragmites* management treatments were randomly assigned to individual 20m x 50m experimental plots such that each treatment was applied to five plots, and no treatment was applied to more than one plot at a single site. Sites were treated as the experimental blocks in ANOVA analyses. This design was necessary due to imazapyr restrictions at one site, and black plastic feasibility concerns at another, preventing us from evaluating all treatments at all sites. Reference plots within native vegetation were also established at each site. Data collected within reference plots were used in multivariate plant community analyses and in figures to provide context, but were not used in ANOVA analysis.


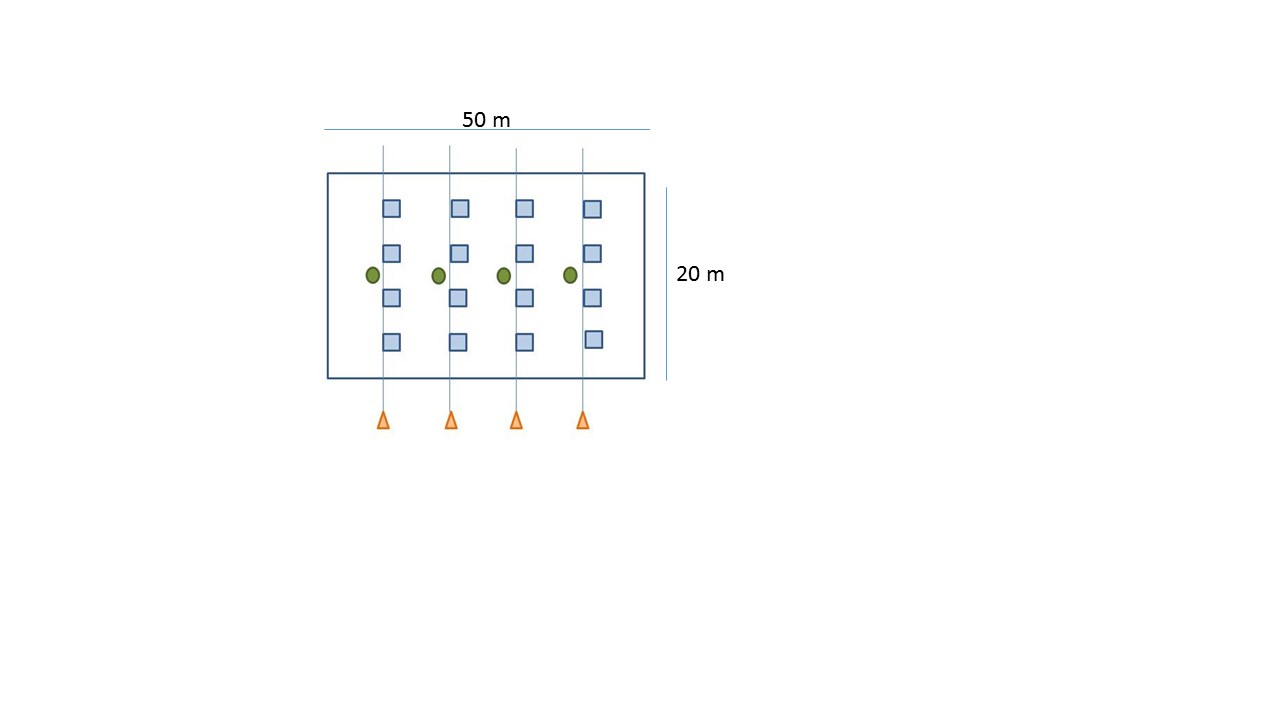


Figure S2. Vegetation and soil sampling diagram for an individual 20m x 50m plot. Vegetation was sampled within 1m^2^ quadrats signified by blue boxes. Quadrats were evenly placed along transects with start points marked by orange triangles. Vegetation was identified to the species level using Flora of Utah (Welsh et al. 1993), and up-to-date nomenclature was determined using USDA PLANTS database (<http://plants.usda.gov>). Soil was sampled at the mid-point of transects, represented by green circles.


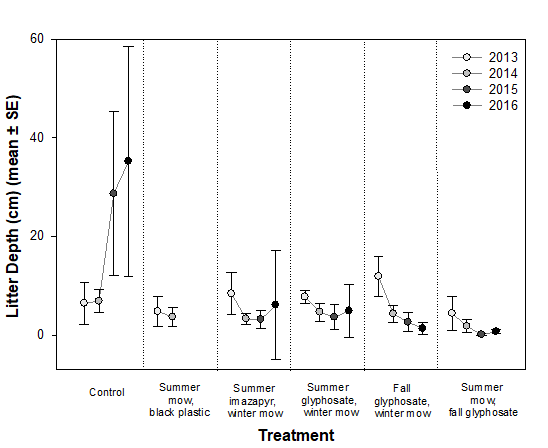


Figure S3. Litter depth following all treatments in summer 2013-2016.

Figure S4. Percent cover of native annuals following each treatment in each year. Pre-treatment data were collected in June 2012, before initial treatments. Follow-up treatments were conducted in 2013 and 2014.
